# Non-invasive assessment of intracranial pressure through the eyes: current developments, limitations, and future directions

**DOI:** 10.3389/fneur.2024.1442821

**Published:** 2024-10-23

**Authors:** Solmaz Bastani Viarsagh, Ashish Agar, Mitchell Lawlor, Clare Fraser, Mojtaba Golzan

**Affiliations:** ^1^Vision Science Group (Orthoptics), Faculty of Health, UTS, Sydney, NSW, Australia; ^2^Ophthalmology Department, Prince of Wales Hospital, UNSW, Darlington, NSW, Australia; ^3^Save Sight Institute, University of Sydney, Sydney, NSW, Australia

**Keywords:** optic nerve sheath diameter, idiopathic intracranial hypertension, spontaneous venous pulsation, vessel analysis, non-invasive and invasive, intracranial pressure, cerebrospinal fluid

## Abstract

Detecting and monitoring elevated intracranial pressure (ICP) is crucial in managing various neurologic and neuro-ophthalmic conditions, where early detection is essential to prevent complications such as seizures and stroke. Although traditional methods such as lumbar puncture, intraparenchymal and intraventricular cannulation, and external ventricular drainage are effective, they are invasive and carry risks of infection and brain hemorrhage. This has prompted the development of non-invasive techniques. Given that direct, non-invasive access to the brain is limited, a significant portion of research has focused on utilizing the eyes, which uniquely provide direct access to their internal structure and offer a cost-effective tool for non-invasive ICP assessment. This review explores the existing non-invasive ocular techniques for assessing chronically elevated ICP. Additionally, to provide a comprehensive perspective on the current landscape, invasive techniques are also examined. The discussion extends to the limitations inherent to each technique and the prospective pathways for future advancements in the field.

## 1 Introduction

A range of neurological and neuro-ophthalmic conditions, including hydrocephalus, space-occupying lesions, and idiopathic intracranial hypertension (IIH), can lead to a chronic rise in the cerebrospinal fluid pressure (CSFp). IIH is characterized by the presence of pathological signs of chronic raised ICP in the absence of any identified dilated ventricles, cranial tumors, or lesions evident on a brain scan with a normal ICP composition (1–4). We use the term CSFp interchangeably with intracranial pressure (ICP) where the latter is in the context of neurological or neuro-ophthalmic disease. Elevated ICP is a critical concern in these conditions, as it can lead to significant morbidity and mortality (5, 6). Rapid detection and management of raised ICP are crucial in preventing further damage to the central nervous system and reducing adverse outcomes. The conventional methods for measuring ICP, such as lumbar puncture (LP) and external ventricular drainage (EVD), are invasive and require trained clinicians to perform (5). These procedures, while effective, are associated with potential complications such as infection, hemorrhage and equipment failure (5, 6).

Headache, nausea, pulsatile tinnitus, and transient vision obscuration have been reported by patients who have elevated ICP (7–9). However, a correlation between the severity of symptoms and the level of elevated ICP has not been found (7, 10). More than 90% of patients complain of throbbing or bursting headaches that can be worsened by sneezing and coughing (2, 7, 11, 12). Loss of consciousness can also occur, which can be due to the displacement of the midbrain and diencephalon (e.g., because of head trauma) or reduction in cerebral perfusion pressure (CPP), leading to decreased cerebral blood flow (13, 14). Other possible clinical signs reported include pupillary dilation, impaired upgaze, and bilateral droopy eyelid (7). Blood pressure and respiratory changes are also often noticed in the late stages of raised ICP, often caused by brain stem distortion and ischemia (7).

In response to the clinical need for safer and more accessible monitoring techniques, non-invasive methods such as transcranial Doppler (TCD), Computed tomography (CT) and Magnetic Resonance imaging (MRI), and non-invasive ophthalmic methods such as the ultrasound assessment of optic nerve sheath diameter (ONSD) have been explored. The TCD methods assesses cerebral blood flow dynamics, which can be altered in the context of raised ICP, while ONSD method is based on the premise that the optic nerve sheath expands in response to elevated ICP. However, studies investigating these non-invasive methods have reported varying degrees of accuracy, suggesting that they may not yet be suitable to replace conventional invasive techniques entirely. Instead, they are often used as complementary tools or to assist in deciding whether to proceed with invasive monitoring (5, 6, 15–18).

## 2 Invasive techniques

Clinical guidelines recommend immediate ICP measurement in patients suspected of elevated ICP (19). However, there is debate concerning the type of pressure being monitored and the optimal location for measuring CSF pressure (20). Currently, raised ICP can be detected invasively at various anatomical locations, including intraventricular, intraparenchymal, epidural, subdural, and subarachnoid sites. LPs are the most commonly used invasive technique to measure and detect raised ICP in acute and chronic conditions (21, 22).

### 2.1 Lumbar puncture

LP is a widely used invasive method for measuring and monitoring chronic and acute raised ICP, such as hydrocephalus and IIH (23). It provides opening and closing CSFp values. Despite its broad use, LP can be a painful procedure and carries risks of infection and brain herniation, however the risks can be reduced by improving skills and techniques (24, 25). Headache is the most common complication reported post lumbar puncture, with up to 32% of the patients reporting some degree of headache (26). Despite the risks associated, LP remains the gold standard technique in measuring and detecting elevated CSFp in acute and chronic raised ICP (27, 28).

### 2.2 Transducer implantation

Various invasive transducer devices have been developed to detect and monitor acute and chronic elevated ICP. These devices involve implanting a catheter connected to a transducer into the brain cavity, with the procedure named after the anatomical location of the insertion site (29). While epidural, subdural, intraparenchymal, and intraventricular methods were previously used, currently only intraventricular, and parenchymal sites are commonly performed (30). The intraparenchymal method measures brain tissue pressure as the catheter is inserted directly into the brain tissue (31, 32). The intraventricular technique was first performed in 1866 by Hollingsworth et al. (30). A catheter is inserted into the ventricles, close to the Foramen of Monroe, through a hole that is drilled into the skull, and connected to an external transducer (20, 29, 30). An External Ventricular Drain (EVD) originating from the intraventricular method is considered the gold standard for measuring global ICP (20, 23, 29, 33–35). An EVD also facilitates CSF drainage to maintain normal ICP levels and allows for the administration of drugs, such as antibiotics (20, 29, 33). The surgical procedure to place an EVD is considered minor and low risk (29). EVD placement can be challenging in younger patients due to their smaller ventricular system, while in older adults, the procedure is relatively straightforward due to age-related ventricular widening (29). A review and meta-analysis reported a 5.7% post-operative risk of hemorrhage in 1790 EVD procedures (36). Infection and colonization of bacteria in the catheter are other complications, potentially leading to skin infection, ventriculitis, meningitis, and septicemia (37–39). Nosocomial infections are the most common complication of EVD, with rates ranging from 5% to over 20% (37). Factors such as catheter misplacement, defective catheters, or skin infection at the insertion site can increase the risk of EVD-related infection (29). A study of 138 patients undergoing EVD found that 12.3% of the catheters were misplaced, necessitating secondary operations (40). EVD is mainly used in acutely raised ICP, such as traumatic brain injury and neurologic intensive care (41, 42).

## 3 Non-invasive assessment of ICP

Due to the complications associated with invasive techniques for measuring ICP, various non-invasive methods have been developed and trialed; however, none have made it into mainstream clinical practice. Techniques such as Computed Tomography (CT), Magnetic Resonance Imaging (MRI), Transcranial Doppler Ultrasonography (TCD), and Tympanic Membrane Displacement (TMD) have been used to screen for signs of elevated ICP and assess the need for invasive monitoring in both chronic and acute raised ICP.

### 3.1 Neuro-imaging

#### 3.1.1 Computed tomography

CT is a non-invasive modality that can be used to detect elevated ICP by evaluating the integrity of intracranial structures. It provides insights into the morphology of midline shifts, basal cisterns, and ventricles. CT scans are often used in combination with other clinical symptoms to evaluate elevated ICP (43, 44). In a study involving 218 patients with severe head injuries, 74% of patients without effacement of basal cisterns had an ICP of more than 30 mmHg (44). Midline shift and compression or destruction of mesencephalic cisterns as observed on CT scans are the most accurate signs of raised ICP (43, 44). In a review of non-invasive methods of measuring ICP, it was concluded that abnormal cisterns are associated with a 3-fold increased risk of elevated ICP (45). CT scans are valuable non-invasive tools used in chronic and acute raised ICP, but a normal CT scan does not necessarily indicate normal ICP. The major reason for performing a CT scan of the brain in IIH patients is to exclude any other underlying cause leading to raised ICP (46, 47).

#### 3.1.2 Magnetic resonance imaging

MRI scans are used to characterize cerebral vascular circulation for non-invasive ICP assessments (48). The exponential relationship between volume and pressure was introduced by Marmarou et al. (49), demonstrating that compliance and resistance to fluid absorption are key in determining ICP levels. Intracranial elastance, which has a linear relationship with ICP, can be measured using MRI (50). Elastance is defined as the change in volume over the change in pressure (Elastance = Δν/∆p). This volume change is obtained using phase-contrast MRI measuring intracranial vein, arterial, and CSF flows during each cardiac cycle, while the pressure change is derived from CSF velocity extracted from velocity-encoded MRI images (43, 48). Studies have examined MRI’s reliability and accuracy in measuring elevated ICP, with findings ranging from modest to poor repeatability to significant correlations with invasive ICP measurements (48, 51) (Table 1).

**Table 1:** Pooled specificity and sensitivity of findings in neuroimaging in IIH patients.

Findings of neuroimaging in IIH	Pooled specificity (95% CI)	Pooled sensitivity (95% CI)
Empty Stella (47, 50, 129–131)	83% (76–90)	80% (71–89)
Papilledema (47, 50, 131, 132)	99% (97–100)	17% (11–13)
Increased ONSD (47, 50, 131)	89% (85–95)	58% (48–68)
Posterior flattening of the eyeball (47, 50, 131)	98% (96–100)	66% (60–72)
Transverse sinus stenosis (46, 47, 133)	93.5% (84–97)	97% (93–100)
Vertical tortuosity of the optic nerve (46, 47, 131)	90% (85–95)	43% (37–50)
Meningoencephalocele (46, 134)	NA	11% (4–18)

### 3.2 Transcranial Doppler ultrasonography

TCD utilizes the interaction between CSF and cerebral blood flow to assess ICP non-invasively. It assesses the blood flow velocity of cerebral arteries and applies mathematical models for ICP estimation, which includes the TCD-derived pulsatility index reflecting downstream vessel resistance, calculation of cerebral perfusion pressure (CPP), and other mathematical estimations. However, the accuracy of these TCD-based methods varies significantly, as noted in a systematic review (15). TCD provides critical information on the direction and velocity of blood flow within intracranial vessels, which can change due to external pressures like ICP or internal pressures such as arterial blood pressure (15).

Despite its advantages of being cost-effective, portable, and risk-free with high temporal resolution, making it ideal for emergency departments and bedside use, TCD has limitations. It cannot detect abnormal ICP if the cause is an obstruction in CSF circulation rather than a dysfunction in the cerebral arterial system (15). Furthermore, its utility in monitoring raised ICP in patients with idiopathic intracranial hypertension (IIH) has shown conflicted results. Some studies suggest that while TCD, particularly the pulsatility index, correlates with raised ICP, it cannot replace invasive methods and is more suitable for monitoring chronically raised ICP during follow-up sessions (52–54). These complexities highlight the need for cautious application and further research into TCD’s effectiveness in various clinical scenarios.

### 3.3 Tympanic membrane displacement

**Figure 1:**
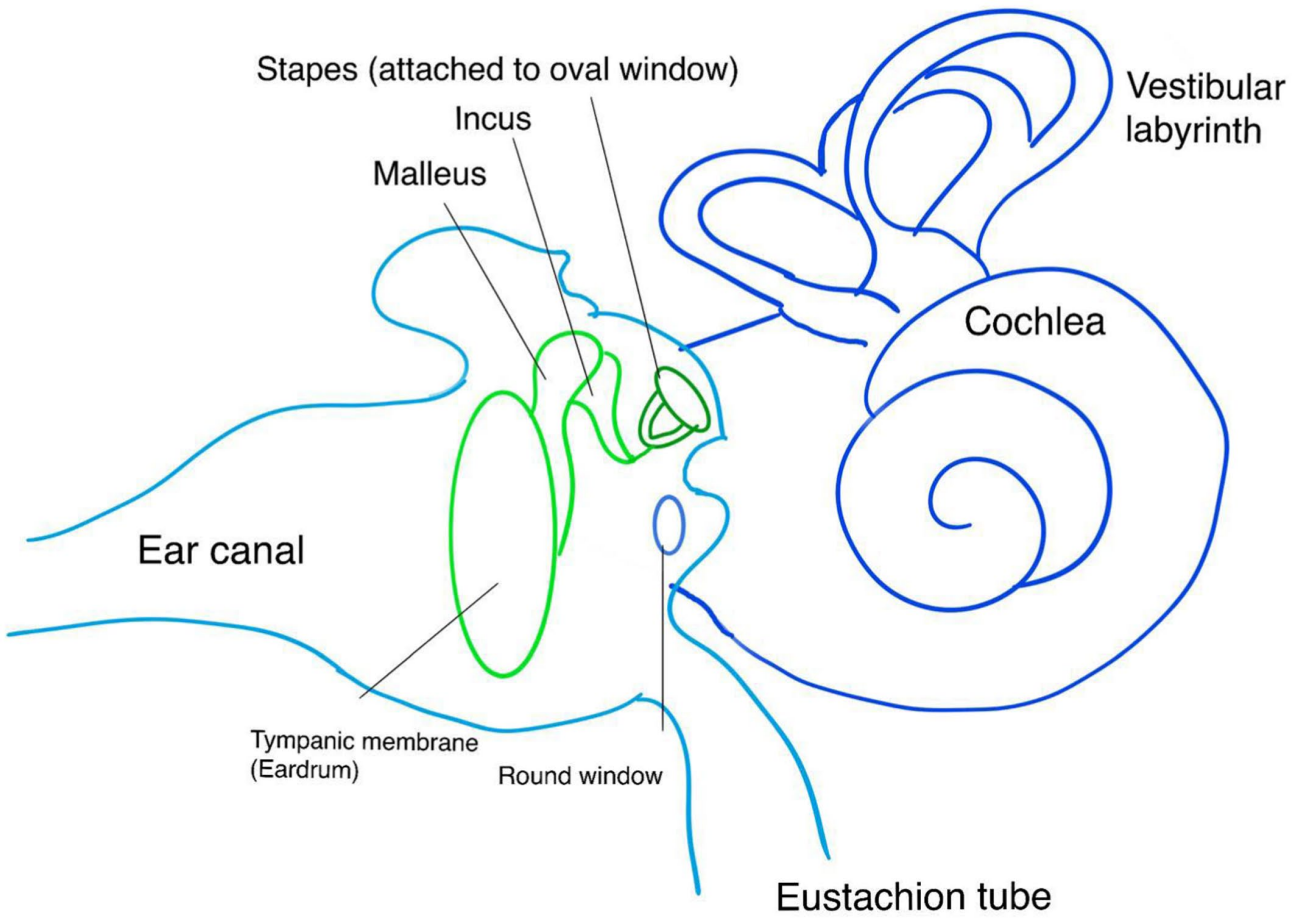
Anatomy of the ear and tympanic membrane involved in the measurement of TMD. CA, Cochlear Aqueduct; OW, Oval Window; RW, Round Window; T, Tympanic membrane; S, Sensor.

TMD, developed in 1981, is an audiological measurement device for detecting raised ICP. It measures the movement of the tympanic membrane in response to the acoustic muscle reflex (55). The stapedius muscle contracts in response to sound stimulation, leading to the movement of the stapes. The cochlear fluid pressure level determines the resting position and direction of stapes movement due to its anatomical position on the oval window. High cochlear fluid pressure results in the inward displacement of the tympanic membrane, while low pressure leads to outward movement. The CSF pressure is transmitted to cochlear fluid pressure through the cochlear aqueduct (Figure 1). Therefore, monitoring the direction of tympanic membrane displacement can indicate normal or raised ICP (39, 56) and it can be an alternative non-invasive method of monitoring raised ICP in Hydrocephalus and neurological conditions (57, 58). To our knowledge, no study in the literature has investigated this method to detect raised ICP in IIH patients.

### 3.4 Otoacoustic emissions

Another novel non-invasive method of detecting elevated ICP is otoacoustic emissions, which is another auditory measurement of acutely elevated ICP. Otoacoustic emissions are produced by cochlea as a response to elevated ICP (59). Distortion product otoacoustic emissions (DPOAE) are subtypes of otoacoustic emissions that are produced in response to two specific frequencies in the ear canal (59). This technique uses microphones placed in the ear canal. Previous studies have shown that as the ICP changes, the magnitude and angles of otoacoustic emissions change; however, the shortcoming of this technique is that small changes in ICP do not produce any systematic changes in DPOAE (59, 60).

### 3.5 Visual evoked potential

**Figure 2:**
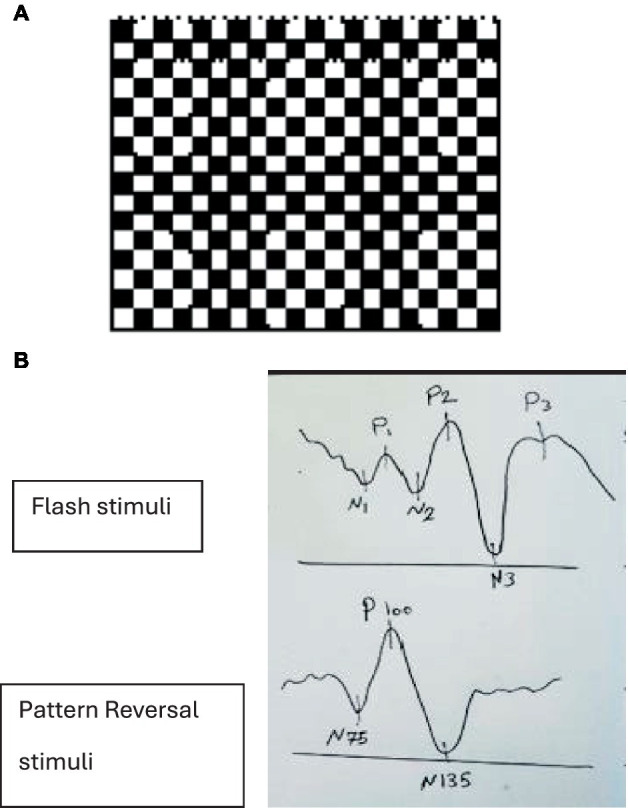
**(A)** Checkboard pattern with a red point fixation target in the middle. **(B)** Positive (P) and negative (N) responses to flash vs. pattern reversal stimuli.

Visual stimuli cause the production of electrical waves in the occipital lobes, which measure the conduction time from the retina to the visual cortex clinically used to examine the integrity of the visual pathway (61). VEP waveforms consist of positive (P) and negative (N) waves, the shape of these waves, the time taken to respond to these stimuli, or the latency (62). In a clinical setting, VEP is obtained by placing electrodes on the patient’s head to measure the optic pathway activity from the retinal pigment epithelium to the occipital lobe (61, 62). This method uses flashes of lights or pattern stimuli; however, flashing VEP (F-VEP) has shown more variability (61). F-VEP lasts for 5 milliseconds, covering a visual field of at least 20 degrees with a recommended flash rate of 1.0 Hz ±0.1 (1 flash per second), and pattern stimuli is a checkboard pattern that reverses every half second (Figure 2A) (63). Previous studies have demonstrated a positive correlation between F-VEP and elevated ICP, mainly causing prolonged latency of N2 waves in patients with traumatic brain injuries, hydrocephalus indicating shunt dysfunction and drug-related cerebral oedema (61, 64–69). Although limited studies are available on the utility of this method in detecting raised ICP in IIH patients, available studies have presented conflicting results depending on the technique used and test time (70). During the early stages of IIH, VEP has been reported to be normal (70–73). However, other studies have shown significant delayed response to stimuli in VEP (74, 75). In conclusion, VEP is useful in monitoring the severity or the regression of the visual function of patients with raised intracranial pressure (76).

Positive (P) and negative (N) responses with different stimuli. In flash stimuli, the second negative peak (N2), with a normal latency of approximately 70–90 ms and the second positive peak (P2), with a normal latency of approximately 100–120 ms, are the most prominent waves. Pattern-reversal VEPs normally comprised of three main features: an initial negativity with a latency of approximately 70 to 80 milliseconds (N75), a larger positive part with a latency of about 90–110 ms (P100), and a large negative part with a latency of around 130–140 ms (N135) (63, 65, 77) (Figure 2B).

## 4 Non-invasive ophthalmic-based assessment

Given the limitations and challenges in directly accessing the brain’s structure, as demonstrated above, alternative ophthalmic-based techniques have been explored due to the anatomical link between the eye and the brain’s structure, vasculature, and CSF drainage pathway. These non-invasive ocular assessments aim to eliminate the need for invasive approaches as the first line of assessment in chronic raised ICP such as IIH. The most commonly studied methods are measuring the optic nerve sheath diameter (ONSD), investigating structural changes at the optic nerve head (papilledema), and examining static and dynamic retinal vessel changes.

### 4.1 Optic nerve sheath diameter

The optic nerve, the second cranial nerve, is responsible for transmitting visual information. It exits the globe at the optic disk, where the lamina cribrosa resides. The optic nerve is surrounded by the optic nerve sheath (ONS), an extension of the brain’s meninges, with CSF running between the optic nerve and the ONS. Early studies have reported that a rise in ICP levels leads to an increase in ONSD, swelling of the optic nerve head, and deformation of the retinal pigment epithelium and Bruch’s membrane in papilledema (78–81). Chronic elevated ICP leads to increased ONSD and increased retinal vein diameter (82).

**Figure 3:**
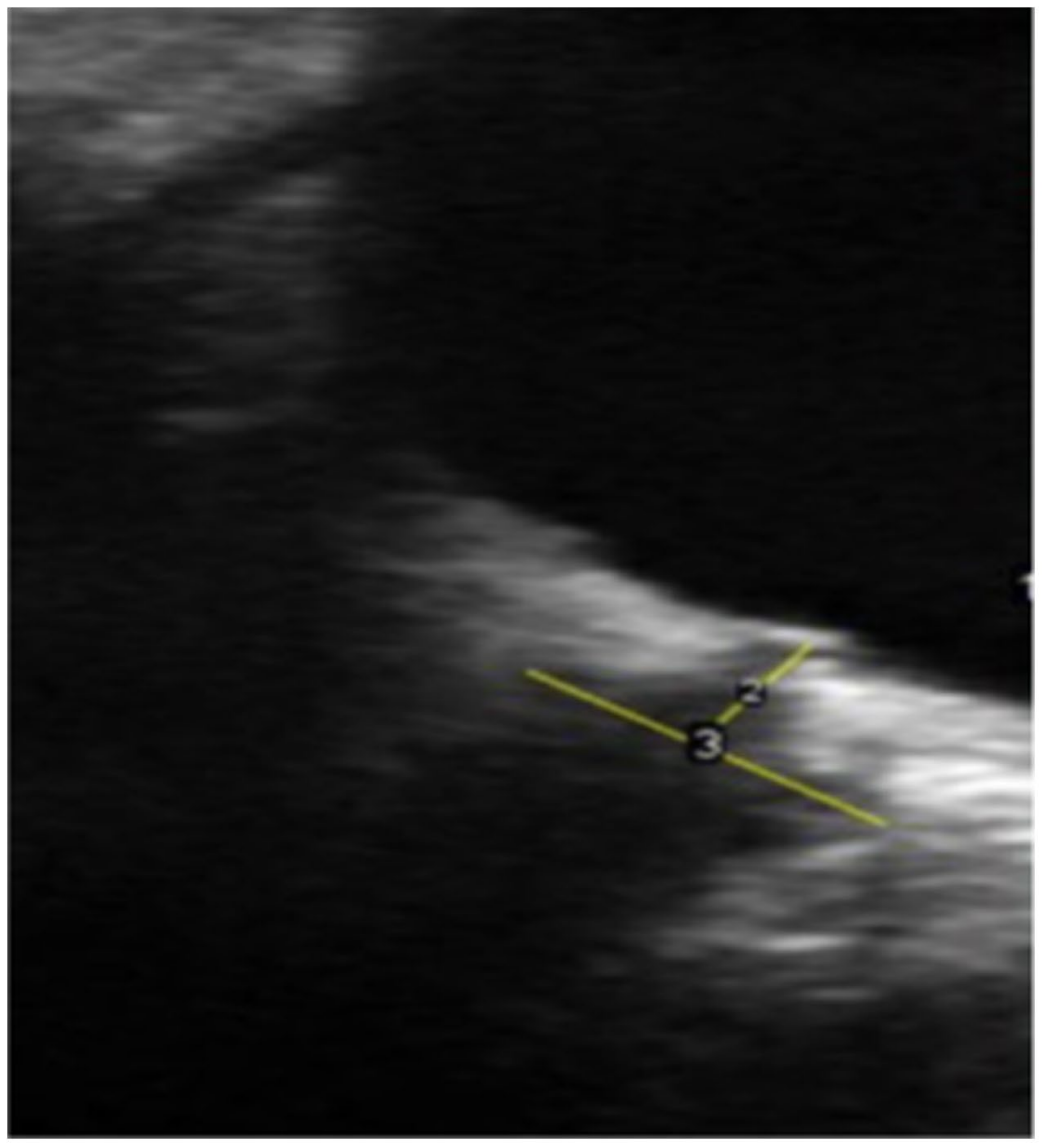
Ultrasound of optic nerve head 3 mm behind the eye socket where optic nerve exits.

Various studies have investigated the correlation between raised ICP and increased ONSD (83–90). Ultrasound is the most investigated non-invasive technique for detecting raised ICP by measuring ONSD, depicted in Figure 1 (91). A study measuring ONSD by ultrasound in 50 participants concluded that an increased diameter of greater than 5.5 mm has 100% specificity and sensitivity in detecting raised ICP of more than 20 cmH2O (86, 91). Previous studies have demonstrated high sensitivity of ONSD in detecting abnormal ICP (83, 85, 87–95). However, there are inconsistent reports in the literature concerning the ONSD cut-off value and the efficacy of tools used to measure ONSD distension in elevated ICP. Cut-off values ranging from 5.3 mm to 5.8 mm have been reported as indicators of raised ICP. Notably, there is considerable inter-individual variation in ONSD measurements. Studies among the Chinese population reported an ONSD threshold of 4.1 mm as a marker of elevated ICP (90), while studies in Western populations reported cut-off values over 5 mm (88–90). In Bangladeshi patients aged over 16, the upper limit of ONSD in healthy individuals is 4.75 mm (96). These variations in cut-off values across different populations lead to increased sensitivity and decreased specificity in estimating raised ICP. Some studies have reported differing findings (85, 90, 95, 96). For example, a study of 51 patients undergoing lumbar puncture showed that ONSD obtained by ultrasound is not an appropriate marker for measuring raised ICP in non-traumatic intracranial hypertension due to its low specificity and sensitivity of 44 and 75%, respectively (85). Conversely, a study examining ICP in patients with severe traumatic brain injury and measuring their ONSD with a CT scan simultaneously reported that assessing ONSD with a portable CT is a reliable method, with a specificity of 42% and sensitivity of 97% (95). Table 2 summarizes the differences in specificity, sensitivity, and cut-off values reported in meta-analyses conducted over the last 10 years (92, 97–103). The variation in findings could be attributed to differences in population ethnicity, sample size, and the modalities used to measure ONSD. Ultrasound of ONSD is highly user-dependent, and measurements can vary significantly depending on the examiner’s skill (Figure 3).

**Table 2:** Specificity, sensitivity, and cut-off value of ONSD in raised ICP, measured non-invasively.

Studies (meta-analysis)	Modality	Pooled specificity (95% CI)	Pooled sensitivity (95% CI)	ONS cut off value ± mean standard deviation (SD)
Aletreby et al. (92)	Ultrasound	0.85 (0.8–0.89)	0.9 (0.85–0.94)	5.55 ± 0.5 mm
Sallam et al. (97)		0.88 (0.84–0.91)	0.9 (0.87–0.92)	Not reported
Lee et al. (98)		0.77 (0.63–0.88)	0.91 (0.87–0.94)	5.8 mm
Koziarz et al. (99)		0.86 (0.74–0.93)	0.97 (0.92–0.99)	5.0 mm
Robba et al. (100)		0.80	0.96	>4.80
Kim et al. (101)		0.73 (0.65–0.8)	0.99 (0.96–1.0)	>5.0 mm
Ohle et al. (102)		0.92 (0.78–0.98)	0.95 (0.87–0.98)	>5.0 mm
Sallam et al. (97)	CT	0.79 (0.56–0.92)	0.93 (0.90–0.96)	Not reported
Sallam et al. (97)	MRI	0.89 (0.84–0.93)	0.77 (0.64–0.87)	Not reported
Kwee et al. (103)		0.86 (0.79–0.91)	0.68 (0.55–0.80)	>2 mm

### 4.2 OCT of the optic nerve head

**Figure 4:**
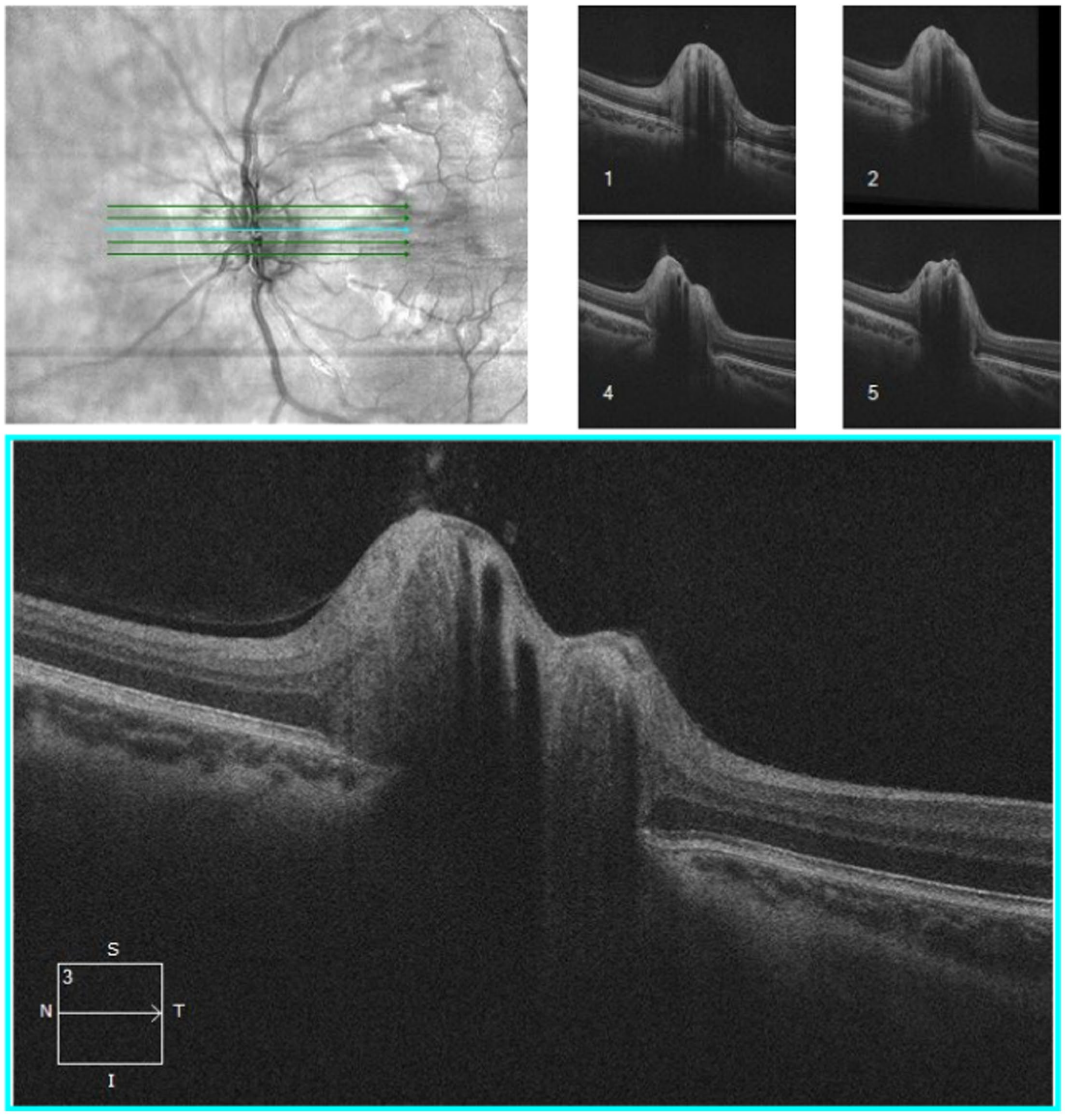
OCT of optic nerve head of an IIH patient conducted by CIRRUS SW Ver: 11.5.2.54532 Copyright 2020 Carl Zeiss Meditec.

Optical coherence tomography (OCT) is a widely used non-invasive technique for diagnosing papilledema, mainly in chronically raised ICP such as IIH. OCT provides cross-sectional images and quantifies retinal nerve fiber thickness (RNFL) and macular ganglion cell layer (GCL), which are key indicators for monitoring papilledema (104). There is thickening of the RNFL in papilledema. The macular GCL thickness is typically normal in patients with preserved visual function. However, in chronic, severe or untreated papilledema, irreversible damage to the retinal ganglion cells is seen as thinning of the macular GCL (105, 106). OCT of optic nerve head (Figure 4) has been instrumental in assessing peripapillary, choroidal, and retinal folds in IIH patients with papilledema, a phenomenon suggested to result from shear stress on the optic nerve head due to raised ICP (107). However, papilledema may not be an optimal marker for diagnosing and monitoring abnormal ICP, as its development and resolution can be delayed, depending on changes in ICP and axoplasmic disturbance. Additionally, optic atrophy may prevent nerve swelling in cases of raised ICP (108). Monitoring the severity of papilledema is suitable for monitoring raised ICP in chronic conditions that may guide treatment (84, 109). Previous studies denoted in Table 3 show the various findings of OCT of the optic nerve head in IIH patients with papilledema (104, 110–113). The increase in RNFL thickness and the decrease in macular GCL are correlated with the severity of papilledema and used to diagnose and monitor the progression and regression of raised ICP, mainly in chronic raised ICP (104, 105, 110, 114). Thinning of macular GCL results in visual dysfunction from neural loss.

**Table 3:** OCT findings of ONH in IIH adult patients with papilledema.

Studies	GCL volume (μm) Mean ± SD	RNFL thickness (μm) Mean ± SD	CSF opening pressure (cmH2O) Mean ± SD
Eren et al. (110)	101.72 ± 7.46 (80.38–130.72)	127.19 ± 24.26 (94–204)	30.0 ± 0.33 (25–37)
Kaufhold et al. (113)	Not reported	99.1 ± 18.1 (58.4–155.5)	28.6 ± 0.63 (18–40)
Waisbourd et al. (111)	210 (199–220)	129.75 ± 41.34 (80–214)	32.6 ± 0.69
Rebolleda and Muñoz-Negrete (104)	Not reported	183.3 ± 74.7	Median 29 (25.5–45)
Jacobsen et al. (112)	Not reported	124.2 ± 77.8	13.4 ± 5.6 (overnight monitoring pressure)

### 4.3 Retinal vessel analysis

#### 4.3.1 Static vessel analysis

Static changes in the retinal vasculature include tortuosity, arteriovenous nicking, and variations in vessel caliber. As ICP exceeds 20 cmH2O, retinal vein diameter increases, resulting in a decreased arteriole-to-venule diameter ratio (115). A study of 20 adults with papilledema observed a reduction in retinal vein and arterial diameter one-hour post-ICP lowering (82). Retinal vein diameter has been proposed as a biomarker for detecting increased retinal vein pressure due to raised ICP (116). In a randomized controlled trial involving 115 individuals with IIH, optic nerve swelling was correlated with increased retinal vein diameter, which decreased 6 months post-treatment for raised ICP (117). Retinal vessel tortuosity, associated with chronic raised ICP, is believed to result from increased vessel diameter and subsequent tortuosity (117, 118) however, no study has systematically investigated the improvement of vessel tortuosity due to the resolution of papilledema. Investigating static vessel changes is mainly used as additional

Markers for diagnosis and monitoring chronic raised ICP.

#### 4.3.2 Dynamic vessel analysis

Dynamic retinal vascular changes are characterized by spatial–temporal features extracted from retinal videos, including pulse wave velocity, arterial pulsatility index, and spontaneous venous pulsations (SVPs) (119–123). SVPs, observed as slight variations in retinal vein diameter at the optic nerve head, occur as a result of intra- and extra-ocular pressure compartments intersecting at the lamina cribrosa, affecting the pressure gradient (120, 122). Elevated ICP leads to increased intracranial pulse pressure and an imbalanced pressure gradient between IOP and ICP, resulting in the cessation of SVPs (122, 124). SVPs have been postulated as markers of raised ICP due to their physiological relationship with ICP and IOP (118–124). A study of 338 measurements from 9 patients showed that ICP pulse pressure controls the timing of venous pulsation (121). While earlier studies reported increased pulsation of the central retinal vein due to increased IOP (125), recent modeling studies have indicated a close relationship between SVP, IOP, and ICP, with any imbalance disrupting SVP oscillation (118, 120, 121).

##### 4.3.2.1 Assessment of SVP

The validity and usefulness of SVPs as a predictive marker of raised ICP has been debated (122–124, 126). The presence of SVPs indicates normal ICP, but their absence does not necessarily signify raised ICP, as they can be absent in 10-20% of the population (122). A prospective study evaluating SVP and ICP in 106 patients undergoing LP concluded that SVP was not a reliable predictor of ICP, with a sensitivity of 94% to exclude raised ICP (126). However, this study assessed ICP and SVP in different positions, which could affect the results. Another study investigated the validity of smartphone-based video ophthalmoscopy for detecting SVP in 233 patients, finding a sensitivity of 82.77 and 76.82% for two observers, respectively (127) demonstrated in Table 4. However, this study faced challenges like poor video quality and camera shake. Subjective measurement of SVPs can be one of the limitations presented in previous studies however objective measurements have shown 100% detection in healthy individuals (128).

**Table 4:** Responses given by two blind observers were asked to identify SVP on Videos, compared with gold standard identification using a slit lamp (127).

	SVP identified on video		SVP not identified on video	
	Observer 1	Observer 2	Observer 1	Observer 2
SVP present on slit lamp	138	116	23	35
SVP not present on slit lamp	13	38	108	83

**Figure 5:**
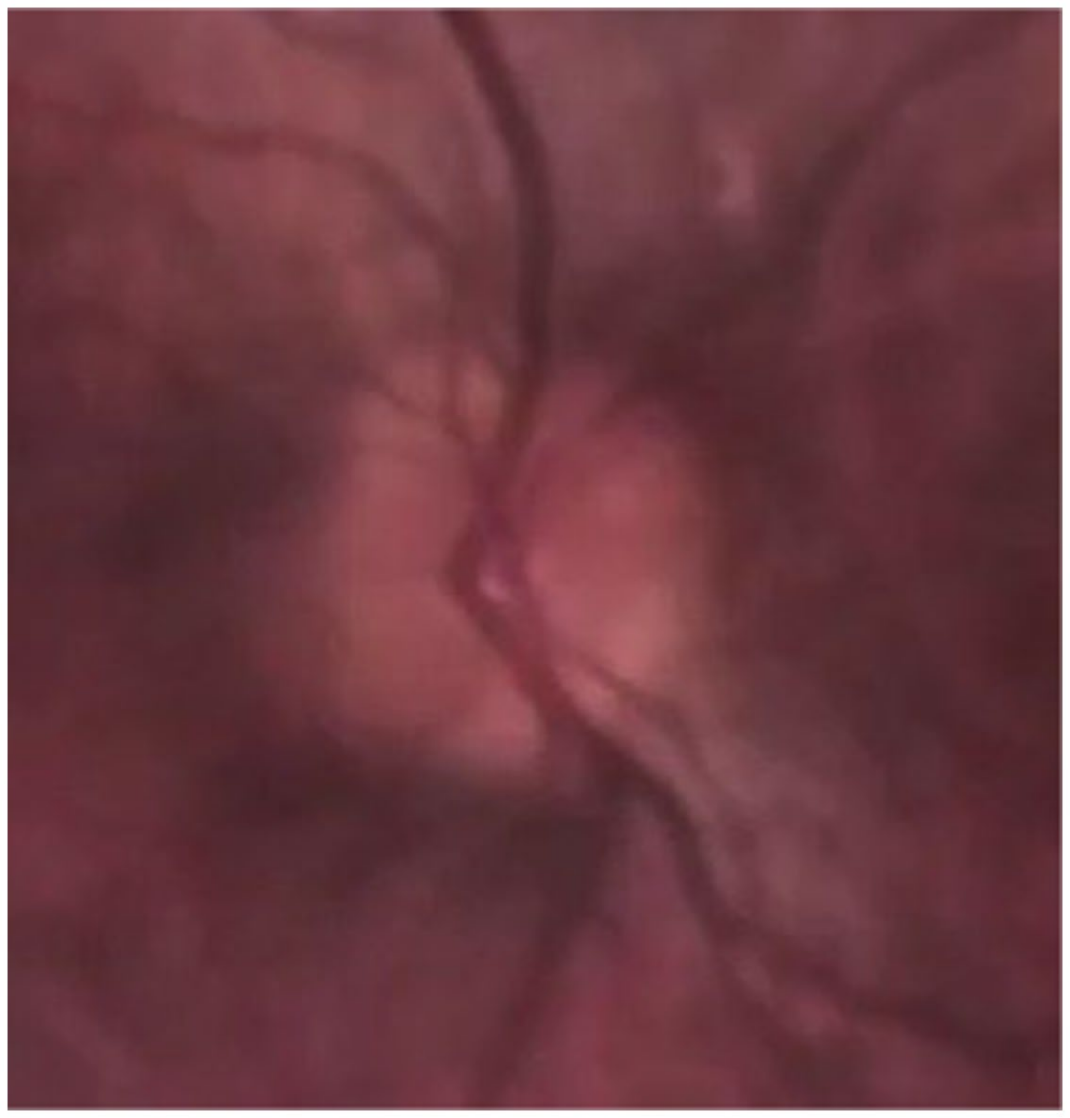
A fundus photo of retinal blood vessels as they exit the globe where SVP is detected with a smartphone-based handheld ophthalmoscope.

A recent study using motion-stabilized infrared videography found a significant association between SVP and ICP in 101 patients with intracranial hypertension (124). This study measured ICP and SVP in a seated position to account for postural variations in ICP (126). While further investigation is needed, there is potential for using SVP to non-invasively and reliably assess ICP (126) (Figure 5).

## 5 Limitations of non-invasive methods

Despite technological advances and the development of various non-invasive techniques, none have yet replaced invasive methods. This is due to limitations such as lack of portability, practicality, and availability in all clinical settings. Additionally, non-invasive methods often cannot monitor ICP continuously or measure it quantitatively, and they tend to be operator-dependent. Currently, non-invasive methods are primarily used as complementary tools in detecting raised ICP.

### 5.1 Neuro-imaging

MRI and CT scans provide qualitative information about structural brain changes, such as midline shift, lateral ventricular compression, and dilation of the ventricular system. These neuro-imaging methods are essential for differentiating underlying conditions from secondary causes of raised ICP. However, they are not universally available. MRI and CT scans cannot measure ICP quantitatively and are not reliable for continuous ICP monitoring. MRI is time-consuming, limiting its use in emergency settings where rapid detection of raised ICP is crucial. CT scans, which use ionizing radiation, are particularly concerning in pediatric patients. Collectively, neuro-imaging techniques are primarily used as screening tools where available.

### 5.2 Tympanic membrane displacement and otoacoustic emissions

The TMD technique requires a normal middle ear, an intact acoustic stapedial reflex, and a patent cochlear aqueduct. It is less optimal in elderly patients, as cochlear aqueduct patency often decreases with age. TMD cannot measure absolute ICP but can provide mean ICP values; however, it is not suitable for continuous ICP monitoring. Despite these limitations, TMD has proven reliable for screening patients suspected of having raised ICP. Otoacoustic emissions do not show any changes if the elevation of ICP is minor, which makes this method unreliable in acute smaller changes of ICP.

### 5.3 VEP

This non-invasive method is useful in detecting abnormalities in visual pathway dysfunction based on raised ICP. VEP cannot measure absolute ICP as it only detects damage to the optic nerve. If the patient is at an early stage of the disease and optic nerve damage has not happened yet, then the diagnosis can be missed or remain undetected. Another point to consider is that VEP is affected by anesthesia and cannulation and cannot be used in patients who are under anesthesia. However, it can be used as a non-invasive way of monitoring the progression of raised ICP in chronic intracranial hypertension patients.

### 5.4 Ophthalmic-based assessment

Ocular-based technologies and methods for non-invasive assessment of ICP have made significant advancements but have not yet achieved the clinical reliability needed to replace invasive methods. Limitations include structural variations in the eye, such as ONSD, across different ethnicities. Additionally, ocular changes may not be clinically sensitive to detect subtle, temporary ICP changes. An increase in ICP must be sustained and significant to affect retinal structures, including papilledema and blood vessel changes like the cessation of SVPs. Furthermore, none of the non-invasive ophthalmic methods developed so far can measure mean ICP; they assess abnormalities in retinal and optic nerve head structure and vasculature as surrogate biomarkers. Quantitative measurement of retinal and cranial pulse pressure also remains challenging.

## 6 Future directions

Developing an accurate, non-invasive method for monitoring elevated ICP could significantly impact patient quality of life. Traditional invasive methods, such as lumbar puncture, external ventricular drainage, and intraparenchymal probes, carry risks of complications like infection and brain herniation. The non-invasive techniques developed to date have their shortcomings and do not provide precise measurements of raised ICP. A holistic understanding of the link between ICP and structural and vascular changes is necessary to better comprehend the pathophysiology of raised ICP and its effects on retinal structures and blood vessels. We propose that elevated ICP could be monitored using a combination of non-invasive parameters, such as SVP, OCT of RNFL, and fundus photos, to establish a panel of biomarkers. In taking such approach a versatile panel of biomarkers can be tuned for detecting both acute and chronic elevations in ICP. For chronic conditions, more structural changes are expected, making markers such as RNFL assessments or static vascular features particularly relevant. In contrast, acute conditions, due to their dynamic nature, may show more prominent changes in dynamic vascular features (e.g., SVPs), which could more accurately reflect acute elevations in ICP. This does not imply that certain manifestations are exclusive to one condition over the other but suggests that the likelihood of observing a change may be more pronounced in one type of feature compared to another. Collectively, this approach could enhance the specificity and sensitivity of using ophthalmic-based markers to assess raised ICP non-invasively. While this research might lead to superior non-invasive ICP assessment methods through ocular examination, it is unlikely to replace gold-standard invasive techniques but rather act as a complementary approach where access to invasive techniques is limited or not warranted. It could also be valuable for early screening and triaging, thereby improving clinical management.

## References

[1] Raichle ME Grubb RL Phelps ME Gado MH Caronna JJ. Cerebral hemodynamics and metabolism in Pseudotumor cerebri. Ann Neurol. (1978) 4:104–11. doi: 10.1002/ana.410040203 707980

[2] Lueck CJ McIlwaine GG. Interventions for idiopathic intracranial hypertension In: Lueck CJ, editor. Cochrane database of systematic reviews. Chichester: John Wiley & Sons, Ltd (2005) 10.1002/14651858.CD003434.pub2 16034899

[3] Kesler A Gadoth N. Epidemiology of idiopathic Intracranial hypertension in Israel. J Neuroophthalmol. (2001) 21:12–4. doi: 10.1097/00041327-200103000-00003, PMID: 11315973

[4] Mollan SP Aguiar M Evison F Frew E Sinclair AJ. The expanding burden of idiopathic intracranial hypertension. Eye. (2019) 33:478–85. doi: 10.1038/s41433-018-0238-5, PMID: 30356129 PMC6460708

[5] Changa AR Czeisler BM Lord AS. Management of elevated intracranial pressure: a review. Curr Neurol Neurosci Rep. (2019) 19:99. doi: 10.1007/s11910-019-1010-3 31773291

[6] Schizodimos T Soulountsi V Iasonidou C Kapravelos N. An overview of management of intracranial hypertension in the intensive care unit. J Anesth. (2020) 34:741–57. doi: 10.1007/s00540-020-02795-7, PMID: 32440802 PMC7241587

[7] Dunn LT. Raised intracranial pressure. J Neurol Neurosurg Psychiatry. (2002) 73:i23–7. doi: 10.1136/jnnp.73.suppl_1.i23, PMID: 12185258 PMC1765599

[8] Qvarlander S Sundström N Malm J Eklund A. Postural effects on intracranial pressure: modeling and clinical evaluation. J Appl Physiol. (2013) 115:1474–80. doi: 10.1152/japplphysiol.00711.2013, PMID: 24052030

[9] Marmarou A Shulman K Rosende RM. A nonlinear analysis of the cerebrospinal fluid system and intracranial pressure dynamics. J Neurosurg. (1978) 48:332–44. doi: 10.3171/jns.1978.48.3.0332, PMID: 632857

[10] Ko MW Chang SC Ridha MA Ney JJ Ali TF Friedman DI. Weight gain and recurrence in idiopathic intracranial hypertension: a case-control study. Neurology. (2011) 76:1564–7. doi: 10.1212/WNL.0b013e3182190f51, PMID: 21536635

[11] Radojicic A Vukovic-Cvetkovic V Pekmezovic T Trajkovic G Zidverc-Trajkovic J Jensen RH. Predictive role of presenting symptoms and clinical findings in idiopathic intracranial hypertension. J Neurol Sci. (2019) 399:89–93. doi: 10.1016/j.jns.2019.02.006 30782528

[12] Pickard JD Czosnyka M. Management of raised intracranial pressure. J Neurol Neurosurg Psychiatry. (1993) 56:845–58. doi: 10.1136/jnnp.56.8.845, PMID: 8350099 PMC1015137

[13] Balestreri M Czosnyka M Hutchinson P Steiner LA Hiler M Smielewski P. Impact of Intracranial pressure and cerebral perfusion pressure on severe disability and mortality after head injury. Neurocrit Care. (2006) 4:008–13. doi: 10.1385/NCC:4:1:008, PMID: 16498188

[14] Chen H Wang J Mao S Dong W Yang H. A new method of Intracranial pressure Monitoring by EEG power Spectrum analysis. Can J Neurol Sci. (2012) 39:483–7. doi: 10.1017/S0317167100013998, PMID: 22728855

[15] Cardim D Robba C Bohdanowicz M Donnelly J Cabella B Liu X. Non-invasive monitoring of intracranial pressure using transcranial Doppler ultrasonography: is it possible? Neurocrit Care. (2016) 25:473–91. doi: 10.1007/s12028-016-0258-6 26940914 PMC5138275

[16] Aaslid R Markwalder TM Nornes H. Noninvasive transcranial Doppler ultrasound recording of flow velocity in basal cerebral arteries. J Neurosurg. (1982) 57:769–74. doi: 10.3171/jns.1982.57.6.0769, PMID: 7143059

[17] Robba C Cardim D Sekhon M Budohoski K Czosnyka M. Transcranial Doppler: a stethoscope for the brain-neurocritical care use. J Neurosci Res. (2018) 96:720–30. doi: 10.1002/jnr.24148, PMID: 28880397

[18] Fernando SM Tran A Cheng W Rochwerg B Taljaard M Kyeremanteng K. Diagnosis of elevated intracranial pressure in critically ill adults: systematic review and meta-analysis. BMJ. (2019) 366:l4225. doi: 10.1136/bmj.l4225 31340932 PMC6651068

[19] Cook AM Morgan Jones G Hawryluk GWJ Mailloux P McLaughlin D Papangelou A. Guidelines for the acute treatment of cerebral edema in Neurocritical care patients. Neurocrit Care. (2020) 32:647–66. doi: 10.1007/s12028-020-00959-7, PMID: 32227294 PMC7272487

[20] Smith M. Monitoring Intracranial pressure in traumatic brain injury. Anesth Analg. (2008) 106:240–8. doi: 10.1213/01.ane.0000297296.52006.8e, PMID: 18165584

[21] Bothwell SW Janigro D Patabendige A. Cerebrospinal fluid dynamics and intracranial pressure elevation in neurological diseases. Fluids Barriers CNS. 16:9. doi: 10.1186/s12987-019-0129-6 PMC6456952 30967147

[22] Sakka L Coll G Chazal J. Anatomy and physiology of cerebrospinal fluid. Eur Ann Otorhinolaryngol Head Neck Dis. (2011) 128:309–16. doi: 10.1016/j.anorl.2011.03.002, PMID: 22100360

[23] Czosnyka M. Monitoring and interpretation of intracranial pressure. J Neurol Neurosurg Psychiatry. (2004) 75:813–21. doi: 10.1136/jnnp.2003.033126, PMID: 15145991 PMC1739058

[24] Van Crevel H Hijdra A De Gans J. Lumbar puncture and the risk of herniation: when should we first perform CT? J Neurol. (2002) 249:129–37. doi: 10.1007/PL00007855, PMID: 11985377

[25] Sternbach G. Lumbar puncture. J Emerg Med. (1985) 2:199–203. doi: 10.1016/0736-4679(85)90397-X, PMID: 3833922

[26] Armon C Evans RW. Addendum to assessment: prevention of post–lumbar puncture headaches [RETIRED]. Neurology. (2005) 65:510–2. doi: 10.1212/01.wnl.0000173034.96211.1b, PMID: 16116106

[27] Wright BLC Lai JTF Sinclair AJ. Cerebrospinal fluid and lumbar puncture: a practical review. J Neurol. (2012) 259:1530–45. doi: 10.1007/s00415-012-6413-x, PMID: 22278331

[28] Doherty CM Forbes RB. Diagnostic lumbar puncture. Ulster Med J. (2014) 83:93–102. PMID: 25075138 PMC4113153

[29] Raboel PH Bartek J Andresen M Bellander BM Romner B. Intracranial pressure Monitoring: invasive versus non-invasive methods—a review. Crit Care Res Pract. (2012) 2012:1–14. doi: 10.1155/2012/950393, PMID: 22720148 PMC3376474

[30] Olson DM Ortega Peréz S Ramsay J Venkatasubba Rao CP Suarez JI McNett M. Differentiate the source and site of Intracranial pressure measurements using more precise nomenclature. Neurocrit Care. (2019) 30:239–43. doi: 10.1007/s12028-018-0613-x, PMID: 30251073

[31] Ostrup RC Luerssen TG Marshall LF Zornow MH. Continuous monitoring of intracranial pressure with a miniaturized fiberoptic device. J Neurosurg. (1987) 67:206–9. doi: 10.3171/jns.1987.67.2.0206, PMID: 3598682

[32] Reulen HJ Kreysch HG. Measurement of brain tissue pressure in cold induced cerebral oedema. Acta Neurochir. (1973) 29:29–40. 4780647 10.1007/BF01414614

[33] Steiner LA Andrews PJD. Monitoring the injured brain: ICP and CBF. Br J Anaesth. (2006) 97:26–38. doi: 10.1093/bja/ael110, PMID: 16698860

[34] Zhong J Dujovny M Park HK Perez E Perlin AR Diaz FG. Advances in ICP monitoring techniques. Neurol Res. (2003) 25:339–50. doi: 10.1179/016164103101201661 12870259

[35] March K. Intracranial pressure monitoring. AACN Clin Issues. (2005) 16:456–75. doi: 10.1097/00044067-200510000-00004 16269892

[36] Binz DD Toussaint LG Friedman JA. Hemorrhagic complications of Ventriculostomy placement: a Meta-analysis. Neurocrit Care. (2009) 10:253. doi: 10.1007/s12028-009-9193-0 19224404

[37] Beer R Lackner P Pfausler B Schmutzhard E. Nosocomial ventriculitis and meningitis in neurocritical care patients. J Neurol. (2008) 255:1617–24. doi: 10.1007/s00415-008-0059-8, PMID: 19156484

[38] Lozier AP Sciacca RR Romagnoli MF Connolly ES. Ventriculostomy-related infections: a critical review of the literature. Neurosurgery. (2002) 51:170–82. doi: 10.1097/00006123-200207000-00024, PMID: 12182415

[39] Reid A Marchbanks RJ Bateman DE Martin AM Brightwell AP Pickard JD. Mean intracranial pressure monitoring by a non-invasive audiological technique: a pilot study. J Neurol Neurosurg Psychiatry. (1989) 52:610–2. doi: 10.1136/jnnp.52.5.610, PMID: 2732731 PMC1032174

[40] Saladino A White JB Wijdicks EFM Lanzino G. Malplacement of ventricular catheters by neurosurgeons: a single institution experience. Neurocrit Care. (2009) 10:248–52. doi: 10.1007/s12028-008-9154-z, PMID: 18923816

[41] Muralidharan R. External ventricular drains: management and complications. Surg Neurol Int. (2015) 6:271. doi: 10.4103/2152-7806.157620, PMID: 26069848 PMC4450504

[42] Chau CYC Craven CL Rubiano AM Adams H Tülü S Czosnyka M. The evolution of the role of external ventricular drainage in traumatic brain injury. J Clin Med. (2019) 8:1422. doi: 10.3390/jcm8091422, PMID: 31509945 PMC6780113

[43] Kristiansson H Nissborg E Bartek J Andresen M Reinstrup P Romner B. Measuring elevated Intracranial pressure through noninvasive methods. J Neurosurg Anesthesiol. (2013) 25:372–85. doi: 10.1097/ANA.0b013e31829795ce, PMID: 23715045

[44] Toutant SM Klauber MR Marshall LF Toole BM Bowers SA Seelig JM. Absent or compressed basal cisterns on first CT scan: ominous predictors of outcome in severe head injury. J Neurosurg. (1984) 61:691–4. doi: 10.3171/jns.1984.61.4.0691, PMID: 6470778

[45] Rosenberg JB Shiloh AL Savel RH Eisen LA. Non-invasive methods of estimating intracranial pressure. Neurocrit Care. (2011) 15:599–608. doi: 10.1007/s12028-011-9545-4 21519957

[46] Bidot S Saindane AM Peragallo JH Bruce BB Newman NJ Biousse V. Brain imaging in idiopathic Intracranial hypertension. J Neuroophthalmol. (2015) 35:400–11. doi: 10.1097/WNO.0000000000000303, PMID: 26457687

[47] Maralani PJ Hassanlou M Torres C Chakraborty S Kingstone M Patel V. Accuracy of brain imaging in the diagnosis of idiopathic intracranial hypertension. Clin Radiol. (2012) 67:656–63. doi: 10.1016/j.crad.2011.12.002, PMID: 22309765

[48] Alperin NJ Lee SH Loth F Raksin PB Lichtor T. MR-Intracranial pressure (ICP): a method to measure Intracranial Elastance and pressure noninvasively by means of MR imaging: baboon and human study. Radiology. (2000) 217:877–85. doi: 10.1148/radiology.217.3.r00dc42877, PMID: 11110957

[49] Marmarou A Shulman K LaMorgese J. Compartmental analysis of compliance and outflow resistance of the cerebrospinal fluid system. J Neurosurg. (1975) 43:523–34. 1181384 10.3171/jns.1975.43.5.0523

[50] Ridha MA Saindane AM Bruce BB Riggeal BD Kelly LP Newman NJ. Magnetic resonance imaging findings of elevated Intracranial pressure in cerebral venous thrombosis versus idiopathic Intracranial hypertension with transverse sinus stenosis. Neuro-Ophthalmology. (2013) 37:1–6. doi: 10.3109/01658107.2012.738759, PMID: 24019557 PMC3765015

[51] Marshall I Maccormick I Sellar R Whittle I. Assessment of factors affecting MRI measurement of intracranial volume changes and elastance index. Br J Neurosurg. (2008) 22:389–97. doi: 10.1080/02688690801911598 18568727

[52] Hunter G Voll C Rajput M. Utility of transcranial Doppler in idiopathic Intracranial hypertension. Can J Neurol Sci. (2010) 37:235–9. doi: 10.1017/S0317167100009987, PMID: 20437935

[53] Gur AY Kesler A Shopin L Bornstein NM. Transcranial Doppler for evaluation of idiopathic intracranial hypertension. Acta Neurol Scand. (2007) 116:239–42. doi: 10.1111/j.1600-0404.2007.00861.x, PMID: 17824902

[54] Pradeep R Gupta D Shetty N Bhushan AK Haskar K Gogineni S. Transcranial Doppler for Monitoring and evaluation of idiopathic Intracranial hypertension. J Neurosci Rural Pract. (2020) 11:309–14. doi: 10.1055/s-0040-1710086, PMID: 32405187 PMC7214091

[55] Marchbanks RJ. Measurement of tympanic membrane displacement arising from aural cardiovascular activity, swallowing, and intra-aural muscle reflex. Acta Otolaryngol. (1984) 98:119–29. 6464714 10.3109/00016488409107543

[56] Samuel M Burge DM Marchbanks RJ. Tympanic membrane displacement testing in regular assessment of intracranial pressure in eight children with shunted hydrocephalus. J Neurosurg. (1998) 88:983–95. doi: 10.3171/jns.1998.88.6.0983, PMID: 9609292

[57] Reid A Marchbanks RJ Burge DM Martin AM Bateman DE Pickard JD. The relationship between intracranial pressure and tympanic membrane displacement. Br J Audiol. (1990) 24:123–9. 2350622 10.3109/03005369009077853

[58] Campbell-Bell CM Birch AA Vignali D Bulters D Marchbanks RJ. Reference intervals for the evoked tympanic membrane displacement measurement: a non-invasive measure of intracranial pressure. Physiol Meas. (2018) 39:015008. doi: 10.1088/1361-6579/aaa1d3, PMID: 29239860

[59] Bershad EM Urfy MZ Pechacek A McGrath M Calvillo E Horton NJ. Intracranial pressure modulates distortion product Otoacoustic emissions. Neurosurgery. (2014) 75:445–55. doi: 10.1227/NEU.0000000000000449, PMID: 24871147

[60] Voss SE Adegoke MF Horton NJ Sheth KN Rosand J Shera CA. Posture systematically alters ear-canal reflectance and DPOAE properties. Hear Res. (2010) 263:43–51. doi: 10.1016/j.heares.2010.03.003 20227475 PMC3179977

[61] MACS V MAS C Costa DL Eulálio KD Vale OC CPB V. Visual evoked potentials show strong positive association with intracranial pressure in patients with cryptococcal meningitis. Arq Neuropsiquiatr. (2015) 73:309–13. doi: 10.1590/0004-282X20150002, PMID: 25992521

[62] Xu W Gerety P Aleman T Swanson J Taylor J. Noninvasive methods of detecting increased intracranial pressure. Childs Nerv Syst. (2016) 32:1371–86. doi: 10.1007/s00381-016-3143-x, PMID: 27351182

[63] Drislane FW. Visual evoked potentials In: The clinical neurophysiology primer. Totowa, NJ: Humana Press. 461–73.

[64] York D Legan M Benner S Watts C. Further studies with a noninvasive method of Intracranial pressure estimation. Neurosurgery. (1984) 14:456–61. doi: 10.1227/00006123-198404000-00011, PMID: 6728148

[65] York DH Pulliam MW Rosenfeld JG Watts C. Relationship between visual evoked potentials and intracranial pressure. J Neurosurg. (1981) 55:909–16. doi: 10.3171/jns.1981.55.6.0909, PMID: 7299465

[66] Stone JL Ghaly RF Hughes JR. Evoked potentials in head injury and states of increased Intracranial pressure. J Clin Neurophysiol. (1988) 5:135–60. doi: 10.1097/00004691-198804000-00002, PMID: 3074972

[67] Sjöström A Uvebrant P Roos A. The light-flash-evoked response as a possible indicator of increased intracranial pressure in hydrocephalus. Childs Nerv Syst. (1995) 11:381–7. doi: 10.1007/BF00717400 7585664

[68] Desch LW. Longitudinal stability of visual evoked potentials in children and adolescents with hydrocephalus. Dev Med Child Neurol. (2001) 43:113. doi: 10.1017/s0012162201000196 11221898

[69] StuartG C Cochrane DD. Visual evoked potentials, intracranial pressure and ventricular size in hydrocephalus. Doc Ophthalmol. (1987) 66:321–9. doi: 10.1007/BF00213660, PMID: 3428086

[70] Kesler A Vakhapova V Korczyn AD Drory VE. Visual evoked potentials in idiopathic intracranial hypertension. Clin Neurol Neurosurg. (2009) 111:433–6. doi: 10.1016/j.clineuro.2008.12.008, PMID: 19345474

[71] Bobak P. Visual evoked potentials to multiple temporal frequencies. Arch Ophthalmol. (1988) 106:936. doi: 10.1001/archopht.1988.01060140082029, PMID: 3390057

[72] Falsini B Tamburrelli C Porciatti V Anile C Porrello G Mangiola N. Pattern Electroretinograms and visual evoked potentials in idiopathic Intracranial hypertension. Ophthalmologica. (1992) 205:194–203. doi: 10.1159/000310341, PMID: 1484689

[73] Verplanck M Kaufman DI Parsons T Yedavally S Kokinakis D. Electrophysiology versus psychophysics in the detection of visual loss in pseudotumor cerebri. Neurology. (1988) 38:1789–9. doi: 10.1212/WNL.38.11.1789, PMID: 3185916

[74] Onofrj M Bodis-Wollner I Mylin L. Visual evoked potential latencies in papilledema and hydrocephalus. Neuro-Ophthalmology. (1981) 2:85–92.

[75] Sorensen PS Trojaborg W Gjerris F Krogsaa B. Visual evoked potentials in Pseudotumor Cerebri. Arch Neurol. (1985) 42:150–3. doi: 10.1001/archneur.1985.04060020064017, PMID: 3977643

[76] Hamamci M Tombul T. Visual evoked potentials follow-up in idiopathic intracranial hypertension. Neurosciences. (2019) 24:185–91. doi: 10.17712/nsj.2019.3.20190004 31380817 PMC8015507

[77] Donnell CP. Visually Evoked Potentials [Internet]. Salt Lake City, UT: University of Utah Health Sciences Center (1995) Webvision: The Organization of the Retina and Visual System; 2012 [cited 2024 Aug 31]. 1–30. Available at: https://www.ncbi.nlm.nih.gov/books/NBK107218/.

[78] Canac N Jalaleddini K Thorpe SG Thibeault CM Hamilton RB. Review: pathophysiology of intracranial hypertension and noninvasive intracranial pressure monitoring. Fluids Barr CNS. (2020) 17:40. doi: 10.1186/s12987-020-00201-8 PMC7310456 32576216

[79] Kupersmith MJ Sibony P Mandel G Durbin M Kardon RH. Optical coherence tomography of the swollen optic nerve head: deformation of the Peripapillary retinal pigment epithelium layer in papilledema. Investigat Opthalmol Vis Sci. (2011) 52:6558. doi: 10.1167/iovs.10-6782, PMID: 21705690 PMC3175986

[80] Hayreh SS. PATHOGENESIS OF OEDEMA OF THE OPTIC DISC (PAPILLOEDEMA). A PRELIMINARY REPORT. Brit J Ophthal [Internet]. (1964) 48:522–43. doi: 10.1136/bjo.48.10.522 14221776 PMC506011

[81] Karam EZ. Optical coherence tomography of the retinal nerve fibre layer in mild papilloedema and pseudopapilloedema. Br J Ophthalmol. (2005) 89:294–8. doi: 10.1136/bjo.2004.049486, PMID: 15722307 PMC1772541

[82] Moss HE Vangipuram G Shirazi Z Shahidi M. Retinal vessel diameters change within 1 hour of Intracranial pressure lowering. Transl Vis Sci Technol [Internet]. (2018) 7:6. doi: 10.1167/tvst.7.2.6, PMID: 29576930 PMC5861929

[83] Onder H Goksungur G Eliacik S Kasim Ulusoy E Arslan G. Neurological research the significance of ONSD, ONSD/ETD ratio, and other neuroimaging parameters in idiopathic intracranial hypertension the significance of ONSD, ONSD/ETD ratio, and other neuroimaging parameters in idiopathic intracranial hypertension. (2021). Available at: https://www.tandfonline.com/action/journalInformation?journalCode=yner20. 10.1080/01616412.2021.1949688 34409925

[84] Huang-Link YM Al-Hawasi A Oberwahrenbrock T Jin YP. OCT measurements of optic nerve head changes in idiopathic intracranial hypertension. Clin Neurol Neurosurg. (2015) 130:122–7. doi: 10.1016/j.clineuro.2014.12.021, PMID: 25614195

[85] Caffery TS Perret JN Musso MW Jones GN. Optic nerve sheath diameter and lumbar puncture opening pressure in nontrauma patients suspected of elevated intracranial pressure. Am J Emerg Med. (2014) 32:1513–5. doi: 10.1016/j.ajem.2014.09.014, PMID: 25284485

[86] Amini A Kariman H Arhami Dolatabadi A Hatamabadi HR Derakhshanfar H Mansouri B. Use of the sonographic diameter of optic nerve sheath to estimate intracranial pressure. Am J Emerg Med. (2013) 31:236–9. doi: 10.1016/j.ajem.2012.06.025, PMID: 22944553

[87] Abdelrahman AS Barakat MMK. MRI measurement of optic nerve sheath diameter using 3D driven equilibrium sequence as a non-invasive tool for the diagnosis of idiopathic intracranial hypertension. Egypt J Radiol Nucl Med. (2020) 51:24. doi: 10.1186/s43055-020-0149-x

[88] Kimberly HH Shah S Marill K Noble V. Correlation of optic nerve sheath diameter with direct measurement of Intracranial pressure. Acad Emerg Med. (2008) 15:201–4. doi: 10.1111/j.1553-2712.2007.00031.x 18275454

[89] Blaivas M Theodoro D Sierzenski PR. BRIEF REPORTS elevated Intracranial pressure detected by bedside emergency ultrasonography of the optic nerve sheath [internet]. Acad Emerg Med. (2003) 10:376–81. doi: 10.1111/j.1553-2712.2003.tb01352.x 12670853

[90] Wang L Feng L Yao Y Wang Y Chen Y Feng J. Optimal optic nerve sheath diameter threshold for the identification of elevated opening pressure on lumbar puncture in a Chinese population. PLoS One. (2015) 10:117939. doi: 10.1371/journal.pone.0119723, PMID: 25664663 PMC4322040

[91] Price DA Grzybowski A Eikenberry J Januleviciene I Verticchio Vercellin AC Mathew S. Review of non-invasive intracranial pressure measurement techniques for ophthalmology applications. Br J Ophthalmol. (2020) 104:887–92. doi: 10.1136/bjophthalmol-2019-314704, PMID: 31704702

[92] Aletreby W Alharthy A Brindley PG Kutsogiannis DJ Faqihi F Alzayer W. Optic nerve sheath diameter ultrasound for raised intracranial pressure a literature review and meta-analysis of its diagnostic accuracy. J Ultrasound Med. (2022) 41:585–95. doi: 10.1002/jum.15732 33893746

[93] Geeraerts T Newcombe VF Coles JP Abate M Perkes IE Hutchinson PJ. Use of T2-weighted magnetic resonance imaging of the optic nerve sheath to detect raised intracranial pressure. Crit Care. (2008) 12:R114. doi: 10.1186/cc7006, PMID: 18786243 PMC2592740

[94] Watanabe A Kinouchi H Horikoshi T Uchida M Ishigame K. Effect of intracranial pressure on the diameter of the optic nerve sheath. J Neurosurg. (2008) 109:255–8. doi: 10.3171/JNS/2008/109/8/0255, PMID: 18671637

[95] Sekhon MS Griesdale DE Robba C McGlashan N Needham E Walland K. Optic nerve sheath diameter on computed tomography is correlated with simultaneously measured intracranial pressure in patients with severe traumatic brain injury. Intensive Care Med. (2014) 40:1267–74. doi: 10.1007/s00134-014-3392-7, PMID: 25034476

[96] Maude RR Hossain A Hassan MU Osbourne S Langan K Sayeed A. Transorbital sonographic evaluation of normal optic nerve sheath diameter in healthy volunteers in Bangladesh. PLoS One. (2013) 8:e81013. doi: 10.1371/journal.pone.0081013 24312515 PMC3846670

[97] Sallam A Alkhatip AAAMM Kamel MG Hamza MK Yassin HM Hosny H. The diagnostic accuracy of noninvasive methods to measure the Intracranial pressure: a systematic review and meta-analysis. Anesth Analg. (2021) 132:686–95. doi: 10.1213/ANE.0000000000005189, PMID: 32991330

[98] Lee SH Kim HS Yun SJ. Optic nerve sheath diameter measurement for predicting raised intracranial pressure in adult patients with severe traumatic brain injury: a meta-analysis. J Crit Care. (2020) 56:182–7. doi: 10.1016/j.jcrc.2020.01.006, PMID: 31945584

[99] Koziarz A Sne N Kegel F Nath S Badhiwala JH Nassiri F. Bedside optic nerve ultrasonography for diagnosing increased Intracranial pressure. Ann Intern Med. (2019) 171:896. doi: 10.7326/M19-0812, PMID: 31739316

[100] Robba C Santori G Czosnyka M Corradi F Bragazzi N Padayachy L. Optic nerve sheath diameter measured sonographically as non-invasive estimator of intracranial pressure: a systematic review and meta-analysis. Intensive Care Med. (2018) 44:1284–94. doi: 10.1007/s00134-018-5305-7, PMID: 30019201

[101] Kim SE Hong EP Kim HC Lee SU Jeon JP. Ultrasonographic optic nerve sheath diameter to detect increased intracranial pressure in adults: a meta-analysis. Acta Radiol. (2019) 60:221–9. doi: 10.1177/0284185118776501, PMID: 29768927

[102] Ohle R McIsaac SM Woo MY Perry JJ. Sonography of the optic nerve sheath diameter for detection of raised Intracranial pressure compared to computed tomography. J Ultrasound Med. (2015) 34:1285–94. doi: 10.7863/ultra.34.7.1285, PMID: 26112632

[103] Kwee RM Kwee TC. Systematic review and meta-analysis of MRI signs for diagnosis of idiopathic intracranial hypertension. Eur J Radiol. (2019) 116:106–15. doi: 10.1016/j.ejrad.2019.04.023, PMID: 31153551

[104] Rebolleda G Muñoz-Negrete FJ. Follow-up of mild papilledema in idiopathic Intracranial hypertension with optical coherence tomography. Investigat Opthalmol Vis Sci. (2009) 50:5197. doi: 10.1167/iovs.08-2528, PMID: 19011007

[105] Scott CJ. Diagnosis and grading of papilledema in patients with raised Intracranial pressure using optical coherence tomography vs clinical expert assessment using a clinical staging scale. Arch Ophthalmol. (2010) 128:705. doi: 10.1001/archophthalmol.2010.94, PMID: 20547947

[106] Malhotra K Padungkiatsagul T Moss HE. Optical coherence tomography use in idiopathic intracranial hypertension. Ann Eye Sci. (2020) 5:7. doi: 10.21037/aes.2019.12.06, PMID: 32405617 PMC7220123

[107] Sibony PA Kupersmith MJ Feldon SE Wang JK Garvin M Auinger P. Retinal and choroidal folds in papilledema. Invest Ophthalmol Vis Sci. (2015) 56:5670–80. doi: 10.1167/iovs.15-17459, PMID: 26335066 PMC4562343

[108] Laemmer R Heckmann JG Mardin CY Schwab S Laemmer AB. Detection of nerve fiber atrophy in apparently effectively treated papilledema in idiopathic intracranial hypertension. Graefes Arch Clin Exp Ophthalmol. (2010) 248:1787–93. doi: 10.1007/s00417-010-1465-z 20677009

[109] Lee A Rigi M Almarzouqi S Morgan M. Papilledema: epidemiology, etiology, and clinical management. Eye Brain. (2015) 7:47. doi: 10.2147/EB.S69174 28539794 PMC5398730

[110] Eren Y Kabatas N Guven H Comoglu S Gurdal C. Evaluation of optic nerve head changes with optic coherence tomography in patients with idiopathic intracranial hypertension. Acta Neurol Belg. (2019) 119:351–7. doi: 10.1007/s13760-018-1000-2, PMID: 30120685

[111] Waisbourd M Leibovitch I Goldenberg D Kesler A. OCT assessment of morphological changes of the optic nerve head and macula in idiopathic intracranial hypertension. Clin Neurol Neurosurg. (2011) 113:839–43. doi: 10.1016/j.clineuro.2011.05.015, PMID: 21700384

[112] Jacobsen HH Jørstad ØK Moe MC Petrovski G Pripp AH Sandell T. Noninvasive estimation of pulsatile and static Intracranial pressure by optical coherence tomography. Transl Vis Sci Technol. (2022) 11:31. doi: 10.1167/tvst.11.1.31, PMID: 35050344 PMC8787623

[113] Kaufhold F Kadas EM Schmidt C Kunte H Hoffmann J Zimmermann H. Optic nerve head quantification in idiopathic Intracranial hypertension by spectral domain OCT. PLoS One. (2012) 7:e36965. doi: 10.1371/journal.pone.0036965, PMID: 22615858 PMC3352870

[114] Skau M Yri H Sander B Gerds TA Milea D Jensen R. Diagnostic value of optical coherence tomography for intracranial pressure in idiopathic intracranial hypertension. Graefes Arch Clin Exp Ophthalmol. (2013) 251:567–74. doi: 10.1007/s00417-012-2039-z, PMID: 22592348

[115] Moss HE Treadwell G Wanek J DeLeon S Shahidi M. Retinal vessel diameter assessment in papilledema by semi-automated analysis of SLO images: feasibility and reliability. Investigat Opthalmol Vis Sci. (2014) 55:2049. doi: 10.1167/iovs.13-13621, PMID: 24609623 PMC3979275

[116] Andersen MS Pedersen CB Poulsen FR. A new novel method for assessing intracranial pressure using non-invasive fundus images: a pilot study. Sci Rep. (2020) 10:13062. doi: 10.1038/s41598-020-70084-0 32747697 PMC7400759

[117] Wall M McDermott MP Kieburtz KD Corbett JJ Feldon SE Friedman DI. Effect of acetazolamide on visual function in patients with idiopathic Intracranial hypertension and mild visual loss. JAMA. (2014) 311:1641. doi: 10.1001/jama.2014.3312, PMID: 24756514 PMC4362615

[118] Moss HE. Retinal vein changes as a biomarker to guide diagnosis and Management of Elevated Intracranial Pressure. Front Neurol. (2021) 12:751370. doi: 10.3389/fneur.2021.751370 34733231 PMC8558235

[119] Morgan WH Hazelton ML Azar SL House PH Yu DY Cringle SJ. Retinal venous pulsation in glaucoma and glaucoma suspects. Ophthalmology. (2004) 111:1489–94. doi: 10.1016/j.ophtha.2003.12.053, PMID: 15288976

[120] Morgan WH Hazelton ML Yu DY. Retinal venous pulsation: expanding our understanding and use of this enigmatic phenomenon. Prog Retin Eye Res. (2016) 55:82–107. doi: 10.1016/j.preteyeres.2016.06.003, PMID: 27417037

[121] Morgan WH Lind CRP Kain S Fatehee N Bala A Yu DY. Retinal vein pulsation is in phase with intracranial pressure and not intraocular pressure. Invest Ophthalmol Vis Sci. (2012) 53:4676–81. doi: 10.1167/iovs.12-9837, PMID: 22700710

[122] Jacks AS Miller NR. Spontaneous retinal venous pulsation: Aetiology and significance. J Neurol Neurosurg Psychiatry. (2003) 74:7–9. doi: 10.1136/jnnp.74.1.7, PMID: 12486256 PMC1738191

[123] Levin BE. The clinical significance of spontaneous pulsations of the retinal vein. Arch Neurol. (1978) 35:37–40. doi: 10.1001/archneur.1978.00500250041009, PMID: 619871

[124] D’Antona L McHugh JA Ricciardi F Thorne LW Matharu MS Watkins LD. Association of intracranial pressure and spontaneous retinal venous pulsation. JAMA Neurol. (2019) 76:1502. doi: 10.1001/jamaneurol.2019.2935, PMID: 31498376 PMC6735496

[125] Engel S. Venous pulsation as a symptom of early Glaucoma*. Am J Ophthalmol. (1946) 29:1446–8. doi: 10.1016/0002-9394(46)92041-7, PMID: 20276980

[126] Wong SH White RP. The clinical validity of the spontaneous retinal venous pulsation. J Neuroophthalmol. (2013) 33:17–20. doi: 10.1097/WNO.0b013e3182622207, PMID: 22801353

[127] Laurent C Hong SC Cheyne KR Ogbuehi KC. The detection of spontaneous venous pulsation with smartphone video ophthalmoscopy. Clin Ophthalmol. (2020) 14:331–7. doi: 10.2147/OPTH.S238897 32099318 PMC7006856

[128] Shariflou S Agar A Rose K Bowd C Golzan SM. Objective quantification of spontaneous retinal venous pulsations using a novel tablet-based ophthalmoscope. Transl Vis Sci Technol. (2020) 9:19. doi: 10.1167/tvst.9.4.19, PMID: 32818106 PMC7396170

[129] Yuh WTC Zhu M Taoka T Quets JP Maley JE Muhonen MG. MR imaging of pituitary morphology in idiopathic intracranial hypertension. J Magn Reson Imaging. (2000) 12:808–13. doi: 10.1002/1522-2586(200012)12:6<808::AID-JMRI3>3.0.CO;2-N, PMID: 11105018

[130] Brodsky M. Magnetic resonance imaging in pseudotumor cerebri. Ophthalmology. (1998) 105:1686–93. doi: 10.1016/S0161-6420(98)99039-X, PMID: 9754178

[131] Agid R Farb RI Willinsky RA Mikulis DJ Tomlinson G. Idiopathic intracranial hypertension: the validity of cross-sectional neuroimaging signs. Neuroradiology. (2006) 48:521–7. doi: 10.1007/s00234-006-0095-y, PMID: 16703359

[132] Saindane AM Bruce BB Riggeal BD Newman NJ Biousse V. Association of MRI findings and visual outcome in idiopathic Intracranial hypertension. Am J Roentgenol. (2013) 201:412–8. doi: 10.2214/AJR.12.9638, PMID: 23883223 PMC4048553

[133] da Silveira Carvalho G de Andrade Matas SL Idagawa MH Tibana LAT de Carvalho RS Silva MLS. A new index for the assessment of transverse sinus stenosis for diagnosing idiopathic intracranial hypertension. J Neurointerv Surg. (2017) 9:173–7. doi: 10.1136/neurintsurg-2016-012605, PMID: 27698231

[134] Lloyd KM DelGaudio JM Hudgins PA. Imaging of Skull Base cerebrospinal fluid leaks in adults. Radiology. (2008) 248:725–36. doi: 10.1148/radiol.2483070362, PMID: 18710972

